# Changes in the expression of proteins associated with aerobic glycolysis and cell migration are involved in tumorigenic ability of two glioma cell lines

**DOI:** 10.1186/1477-5956-10-53

**Published:** 2012-09-03

**Authors:** Anelisa Ramão, Marcela Gimenez, Helen Julie Laure, Clarice Izumi, Rodrigo Cesar dos Santos Vida, Sueli Oba-Shinjo, Suely Kazue Nagahashi Marie, Jose Cesar Rosa

**Affiliations:** 1 Protein Chemistry Center and Department of Molecular and Cell Biology and Pathogenic Bioagents – School of Medicine of Ribeirão Preto, University of São Paulo, Ribeirão Preto, SP, Brazil; 2 Center for Cellular Therapy and Hemotherapy of Ribeirão Preto, Fundação Hemocentro de Ribeirão Preto, Ribeirão Preto, SP, Brazil; 3 Department of Neurology, School of Medicine of São Paulo, University of São Paulo, São Paulo, SP, Brazil

**Keywords:** Glycolysis, Brain cancer, Cell migration, Glioma, Tumorigenesis, U87MG, T98G

## Abstract

**Background:**

The most frequent and malignant brain cancer is glioblastoma multiforme (GBM). In gliomas, tumor progression and poor prognosis are associated with the tumorigenic ability of the cells. U87MG cells (wild-type p53) are known to be tumorigenic in nude mice, but T98G cells (mutant p53) are not tumorigenic. We investigated the proteomic profiling of these two cell lines in order to gain new insights into the mechanisms that may be involved in tumorigenesis.

**Results:**

We found 24 differentially expressed proteins between T98G and U87MG cells. Gene Ontology supports the notion that over-representation of differentially expressed proteins is involved in glycolysis, cell migration and stress oxidative response. Among those associated with the glycolysis pathway, TPIS and LDHB are up-regulated in U87MG cells. Measurement of glucose consumption and lactate production suggests that glycolysis is more effective in U87MG cells. On the other hand, G6PD expression was 3-fold higher in T98G cells and this may indicate a shift to the pentose-phosphate pathway. Moreover, GRP78 expression was also three-fold higher in T98G than in U87MG cells. Under thapsigargin treatment both cell lines showed increased GRP78 expression and the effect of this agent was inversely correlated to cell migration. Quantitative RT-PCR and immunohistochemistry of GRP78 in patient samples indicated a higher level of expression of GRP78 in grade IV tumors compared to grade I and non-neoplastic tissues, respectively.

**Conclusions:**

Taken together, these results suggest an important role of proteins involved in key functions such as glycolysis and cell migration that may explain the difference in tumorigenic ability between these two glioma cell lines and that may be extrapolated to the differential aggressiveness of glioma tumors.

## Background

Gliomas are responsible for more than 60% of all primary brain tumors. Glioblastoma multiforme – GBM, a grade IV tumor (WHO), is one of the most frequent and malignant gliomas [1]. GBM is an aggressive, highly invasive tumor that presents resistance to chemo- and radiotherapy, resulting in a very poor prognosis [2]. Clinical studies have shown improved survival when a combination of radiation and temozolomide chemotherapy is used, but median survival for GBM patients is still about 15 months [3].

U87MG and T98G are malignant glioma cell lines commonly used in functional studies to better understand the biological processes involved in tumor progression, to test drug therapy, or to search for genetic mutations. U87MG is hypo diploid [4] while T98G is a hyperpentaploid cell line [5], a fact that indicates marked changes in their genomes. Moreover, these two cell lines present another major difference: while U87MG cells carry wild-type p53 and are known to be tumorigenic in nude mice [6], T98G cells, harboring the mutant p53, are not tumorigenic. Consequently, p53 mutation alone is inferred not to be sufficient to induce tumorigenesis in nude mice [7]. Nevertheless, the presence of functional wild-type p53 or mutant p53 is taken into account for the genetic sub-classification of GBM as primary or secondary GBM [8,9].

Tumorigenesis is a complex multistep process that involves self-sufficiency in growth signals, insensitivity to growth inhibitory signals, evasion of apoptosis, limitless replicative potential, sustained angiogenesis, tissue invasion, metastasis and two emerging hallmarks recently, added to the list: reprogramming of energy of metabolism and evasion of immune destruction [10]. Invasive and metastatic stages are responsible for most of the mortality of patients with cancer. Tumorigenic ability has also been associated with tumor progression and a poor prognosis in gliomas [11]. Currently, it is unknown which proteins confer the tumorigenic potential observed in U87MG cells. Here we investigated the proteomic profile of U87MG and T98G cell lines and its correlation to proliferation and migration to gain new insights into the mechanisms involved in tumorigenesis.

## Results

### Proteomics analysis: two-dimensional gel electrophoresis

**Figure 1:**
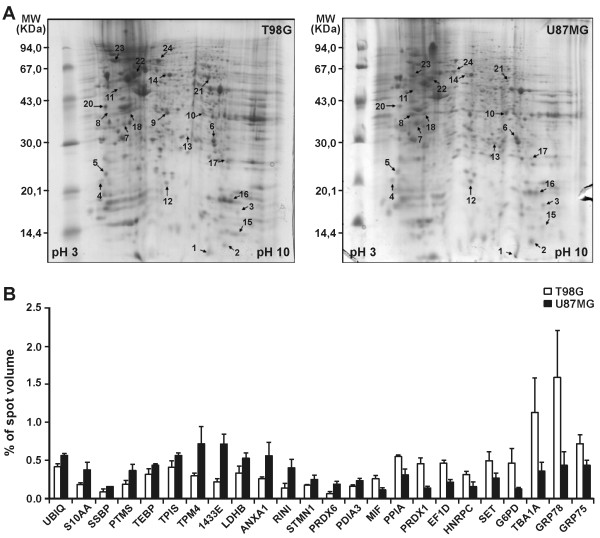
**Representative two-dimensional gel electrophoresis (2-DE) and relative protein abundance plotting of T98G and U87MG cell lines. A**) Protein extracts (300 μg) were applied to 7 cm IPG strips, pH 3-10 non-linear coupled to 12% polyacrylamide gels. Gels were stained using colloidal Coomassie blue. **B**) Differential protein abundance was obtained by normalized spot volumes (% Vol.) and protein identification is reported in Table 1.

U87MG cells (wild-type p53) are known to be tumorigenic in nude mice, but T98G cells (mutant p53) are not tumorigenic. With this in mind, protein extracts of U87MG and T98G cell lines were compared by 2D gel electrophoresis. Analysis of 2DE colloidal Coomassie blue-stained gels detected 386 (SD ± 14) protein spots in T98G cells and 376 (SD ± 35) protein spots in U87MG cells, with molecular masses ranging from 10 to 97 kDa. These similarities in number of protein spots in U87MG and T98G cells suggest that these two cell lines share a common proteome. Representative 2DE gel images from six replicates are shown in Figure 1A. Differentially expressed proteins are labeled in Figure 1B and their identification by mass spectrometry is reported in Table 1. The percentage of spot volumes (% spot vol) was used to measure the protein abundance based on the percent volume of each spot in relation to the sum of all spots detected in the gel. The reproducibility of the gels was determined on the basis of the number of spots that coincided in triplicate gels of two independent experiments. Differential protein abundance was identified when the difference in percent volume of each spot ratio between samples was statistically significant (p < 0.05, Anova).

**Table 1:** Protein identification by MALDI-TOF/TOF-MS of tryptic peptides obtained from 2-DE spots of T98G and U87MG glioma cell lines

**Spot ID**	**U87MG / T98G Expression ratio ± c.v.**	**T98G / U87MG Expression ratio ± c.v.**	**Anova (p value)**	**Accession number (MW / pI)**	**Protein name**	**CID - MS/MS**			
						**Prot score**	**% seq cov**	**Unique peptides**	**Peptide sequence**
1	1.34 ± 0.09	-	0.0053	UBIQ_HUMAN (8565/6.56)	Ubiquitin	302	61.8	1040.24	^34^EGIPPDQQR^42^
								1068.35	^64^ESTLHLVLR^72^
								1082.37	^55^TLSDYNIQK^63^
								1525.84	^30^IQDKEGIPPDQQR^42^
2	1.92 ± 0.34	-	0.0422	S10AA_HUMAN (17259/9.59)	Protein S100-A10	58	10.3	1208.80	^38^EFPGFLENQK^47^
3	1.95 ± 0.52	-	0.0304	SSBP_HUMAN (17259/9.59)	Single-stranded DNA-binding protein, Mitochondrial	263	21.6	986.26	^96^DVAYQYVK^103^
								1150.37	^114^IDYGEYMDK^122^
								1613.00	^67^SGDSEVYQLGDVSQK^81^
4	1.87 ± 0.40	-	0.0356	PTMS_HUMAN (11530/4.14)	Parathymosin	67	10.8	1076.33	^5^SVEAAAELSAK^15^
5	1.37 ± 0.18	-	0.0474	TEBP_HUMAN (18982 / 4.35)	Prostaglandin E synthase 3	230	18.8	922.18	^72^SILCCLR^78^
								1004.22	^80^GESGQSWPR^88^
								1132.42	^79^KGESGQSWPR^88^
								1446.70	^36^LTFSCLGGSDNFK^48^
6	1.39 ± 0.19	-	0.0454	TPIS_HUMAN (26954 / 6.45)	Triosephosphate isomerase	530	34.5	955.17	^7^FVGGNWK^14^
								1138.43	^60^IAVAAQNCYK^69^
								1235.51	^195^SNVSDAVAQSTR^206^
								1327.64	^207^IIYGGSVTGATCK2^19^
								1467.77	^176^TATPQQAQEVHEK^188^
								1587.88	^86^DCGATWVVLGHSER^99^
								1639.98	^70^VTNGAFTGEISPGMIK^85^
7	2.40 ± 0.52	-	0.0351	TPM4_HUMAN (28636 / 4.67)	Tropomyosin alpha-4 chain	253	17.7	1015.23	^45^AEGDVAALNR^54^
								1171.50	^133^LVILEGELER^142^
								1244.53	^56^IQLVEEELDR^65^
								1615.87	^14^IQALQQQADEAEDR^27^
8	3.26 ± 0.47	-	0.0023	1433E_HUMAN (29345 / 4.63)	14-3-3 protein epsilon	519	28.2	908.21	^43^NLLSVAYK^50^
								1206.57	^216^DSTLIMQLLR^225^
								1238.60	^107^HLIPAANTGESK^118^
								1257.58	^131^YLAEFATGNDR^141^
								1385.78	^131^YLAEFATGNDRK^142^
								1448.74	^30^VAGMDVELTVEER^42^
								1837.36	^154^AASDIAMTELPPTHPIR^170^
9	1.57 ± 0.32	-	0.0458	LDHB_HUMAN (36923 / 5.71)	L-lactate dehydrogenase B chain	372	16.8	914.25	^92^IVVVTAGVR^100^
								960.27	^300^GLTSVINQK^308^
								1177.46	^320^SADTLWDIQK^329^
								1249.48	^159^VIGSGCNLDSAR^170^
								1696.03	^8^LIAPVAEEEATVPNNK^23^
10	2.19 ± 0.40	-	0.0330	ANXA1_HUMAN (38942 / 6.57)	Annexin A1	381	13.3	909.05	205ALYEAGER212
								1214.35	167DITSDTSGDFR177
								1263.42	114TPAQFDADELR124
								1740.87	189SEDFGVNEDLADSDAR204
11	3.01 ± 1.08	-	0.0189	RINI_HUMAN(51799 / 4.71)	Ribonuclease inhibitor	579	18.7	1150.41	^35^LDDCGLTEAR^44^
								1161.46	^196^DSPCQLEALK^205^
								1210.51	^54^VNPALAELNLR^64^
								1347.66	^342^FLLELQISNNR^352^
								1514.91	^360^ELCQGLGQPGSVLR^373^
								1531.76	^288^ELSLAGNELGDEGAR^302^
								1631.88	^174^ELTVSNNDINEAGVR^188^
12	1.51 ± 0.17	-	0.0388	STMN1_HUMAN (17302 / 5.76)	Stathmin	219	29.5	1075.39	^44^DLSLEEIQK^52^
								1166.46	^86^AIEENNNFSK^95^
								1327.75	^30^ESVPEFPLSPPK^41^
								1389.81	^15^ASGQAFELILSPR^27^
13	2.99 ± 1.16	-	0.0194	PRDX6_HUMAN (25149 / 6.0)	Peroxiredoxin-6	354	22.8	907.12	^156^NFDEILR^162^
								916.19	^175^VATPVDWK^182^
								1192.53	^145^LSILYPATTGR^155^
								1396.66	^42^DFTPVCTTELGR^53^
								1583.75	^85^DINAYNCEEPTEK^97^
14	1.39 ± 0.14	-	0.0311	PDIA3_HUMAN (57181 / 5.98)	Protein disulfide-isomerase A3	449	15.8	996.28	^131^QAGPASVPLR^140^
								1085.33	^95^YGVSGYPTLK^104^
								1173.40	^336^FVMQEEFSR^344^
								1192.44	^63^LAPEYEAAATR^73^
								1237.40	^108^DGEEAGAYDGPR^119^
								1369.62	^367^SEPIPESNDGPVK^379^
								1681.91	^434^MDATANDVPSPYEVR^448^
15	-	2.33 ± 0.51	0.0099	MIF_HUMAN(12647 / 7.74)	Macrophage migration inhibitory factor	79	17.4	1045.37	79LLCGLLAER87
								1304.69	2PMFIVNTNVPR12
16	-	1.83 ± 0.28	0.0070	PPIA_HUMAN (18240 / 7.68)	Peptidyl-prolyl cis-trans isomerase A	337	32.7	1056.15	20VSFELFADK28
								1155.24	^83^FEDENFILK^91^
								1248.37	^155^KITIADCGQLE^165^
								1295.21	^134^EGMNIVEAMER^144^
								1615.52	^56^IIPGFMCQGGDFTR^69^
17	-	3.62 ± 0.65	0.0022	PRDX1_HUMAN (22338 / 8.27)	Peroxiredoxin-1	178	19.6	921.18	^129^GLFIIDDK^136^
								1108.45	^111^TIAQDYGVLK^120^
								1197.56	^159^LVQAFQFTDK^168^
								1212.59	^141^QITVNDLPVGR^151^
18	-	2.30 ± 0.31	0.0009	EF1D_HUMAN (31236 / 4.9)	Elongation factor 1-delta	458	24.6	974.13	^233^LVPVGYGIR^241^
								1088.16	^39^QENGASVILR^48^
								1359.47	^84^IASLEVENQSLR^95^
								1528.59	^25^FYEQMNGPVAGASR^38^
19	-	2.10 ± 0.60	0.0214	HNRPC_HUMAN (33727 / 4.95)	Heterogeneous nuclearribonucleoproteins C1/C2	123	8.8	904.11	^136^MYSYPAR^142^
								944.28	^143^VPPPPPIAR^151^
								1330.71	^51^GFAFVQYVNER^61^
20	-	1.87 ± 0.47	0.0476	SET_HUMAN(33488 / 4.23)	Protein SET	290	11.7	1209.45	^123^VEVTEFEDIK^132^
								1274.57	^58^LNEQASEEILK^68^
								1447.63	^155^EFHLNESGDPSSK^167^
21	-	3.87 ± 1.03	0.0340	G6PD_HUMAN (59712 / 6.39)	Glucose-6-phosphate1-dehydrogenase	516	15.7	947.20	^10^TQVCGILR^17^
								1003.28	^220^IFGPIWNR^227^
								1059.38	^167^IIVEKPFGR^175^
								1133.39	^321^GYLDDPTVPR^330^
								1174.51	^183^LSNHISSLFR^192^
								1192.42	^499^VGFQYEGTYK^508^
								1233.58	^206^EMVQNLMVLR^215^
								1809.05	^105^NSYVAGQYDDAASYQR^120^
22	-	3.20 ± 1.20	0.0491	TBA1A_HUMAN (50820 / 4.94)	Tubulin alpha-1A chain	390	14.9	904.06	^395^FDLMYAK^401^
								1016.30	^327^DVNAAIATIK^336^
								1086.36	^113^EIIDLVLDR^121^
								1250.36	^312^YMACCLLYR^320^
								1719.98	^216^NLDIERPTYTNLNR229
								1826.21	^353^VGINYQPPTVVPGGDLAK^310^
23	-	3.73 ± 1.52	0.0338	GRP78_HUMAN (72446 / 5.07)	78 kDa glucose-regulated protein	830	20.5	987.10	533LTPEEIER540
								1192.36	^465^VYEGERPLTK^474^
								1212.32	^377^EFFNGKEPSR3^86^
								1218.36	^186^DAGTIAGLNVMR^197^
								1229.35	^50^VEIIANDQGNR^60^
								1317.44	^563^NELESYAYSLK573
								1431.53	^102^TWNDPSVQQDIK^113^
								1461.64	^354^SDIDEIVLVGGSTR^367^
								1567.70	^61^ITPSYVAFTPEGER^74^
								1678.79	^82^NQLTSNPENTVFDAK^96^
								1838.01	^448^SQIFSTASDNQPTVTIK^464^
24	-	1.69 ± 0.29	0.0224	GRP75_HUMAN (73965 / 5.87)	Stress-70 protein, mitochondrial	545	11.5	1243.42	^207^DAGQISGLNVLR^218^
								1291.49	^395^VQQTVQDLFGR^405^
								1362.58	^349^AQFEGIVTDLIR^360^
								1451.59	^86^TTPSVVAFTADGER^99^
								1463.67	^378^SDIGEVILVGGMTR^391^
								1695.86	^188^NAVITVPAYFNDSQR^202^

Fourteen proteins were increased and 10 decreased in abundance in U87MG cells compared to T98G cells. The differentially expressed proteins were classified according to functional gene ontology ( Additional file 1), which showed an over-representation of proteins involved in primary metabolic processes, especially glycolysis, pentose-phosphate shunt, and protein folding. Among the proteins with increased expression in U87MG cells were Annexin A1, Stathmin, 14-3-3E protein, and some enzymes involved in glycolysis, namely TPIS and LDHB. It is also worth to highlight GRP78, involved in protein folding, and G6PD, the first enzyme in the pentose phosphate pathway, which showed 3-fold increased expression in T98G cells compared to U87MG cells.

### Proliferation, migration, glucose consumption and lactate production of T98G and U87MG cells

**Figure 2:**
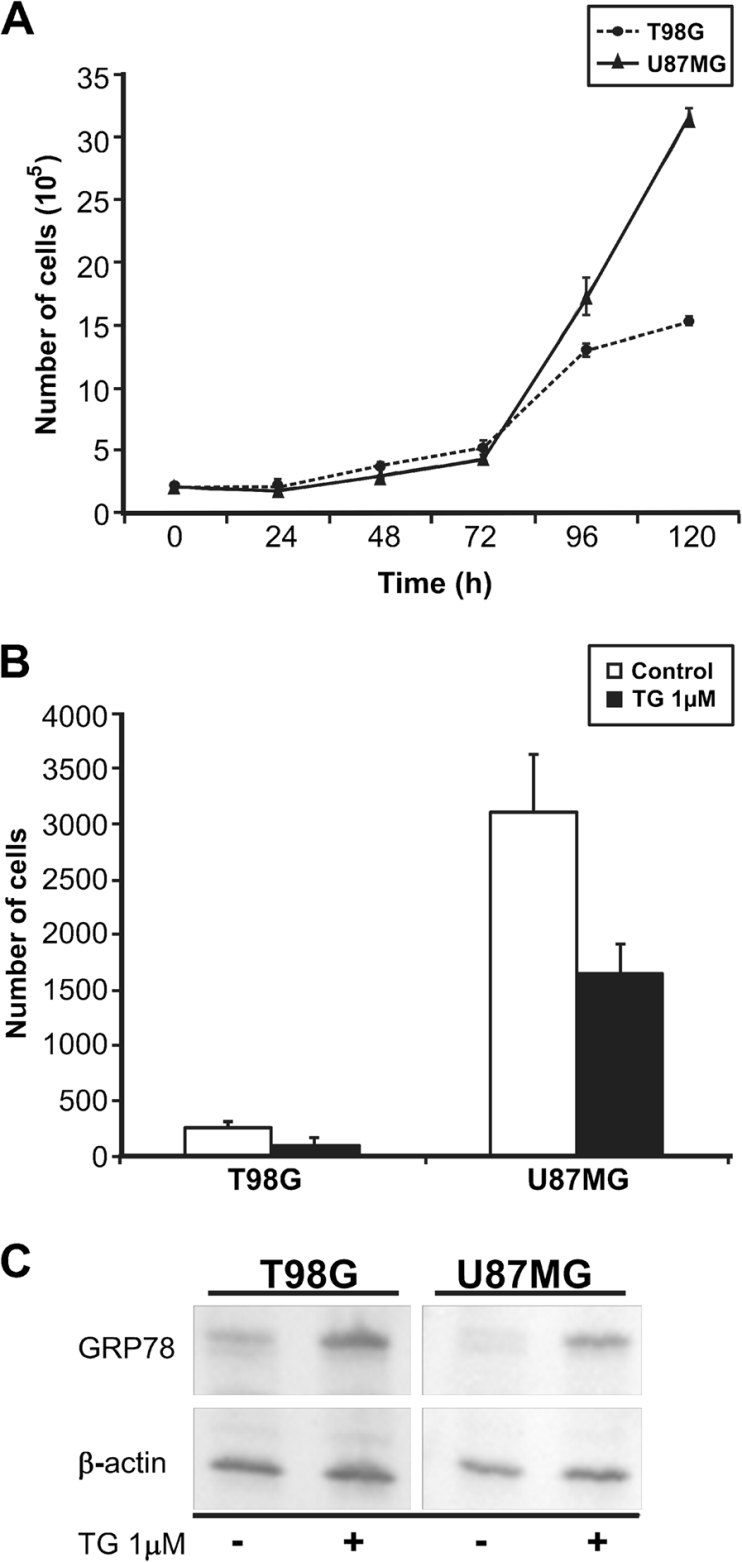
**Functional analysis of cell lineages and expression of GRP78. A**) The U87MG cell line shows a higher proliferation rate than the T98G cell line. Cells were counted in a Neubauer chamber at 24 h intervals; **B**) Thapsigargin decreased tumor cell migration. A transwell migration assay was carried out in U87MG and T98G cells after 18 h of incubation with or without 1 μM thapsigargin. Cells that migrated to the low chamber were fixed, stained, and counted as described in Materials and Methods. **C**) Thapsigargin increased GRP78 expression. Western blot analysis with anti-GRP78 antibody was carried out in both cell lines after 24 h of incubation with 1 μM thapsigargin. Expression of β-actin in cell lysates was used as a control.

Because T98G and U87MG cells differ in their tumorigenic capacity, we looked for differences in their proliferative potential, migration rates, glucose consumption and lactate production. To investigate their proliferation rates, cells were counted in a Neubauer chamber every 24 h. Although in the first 72 h after plating the two cell lines presented similar proliferation rates (Figure 2A), after this period U87MG cells presented a 2-fold higher growth rate compared to T98G cells. However, determination of doubling time between the two cell lines showed very similar results, i.e., 29.2 h for U87MG and 29.9 h for T98G.

**Figure 3:**
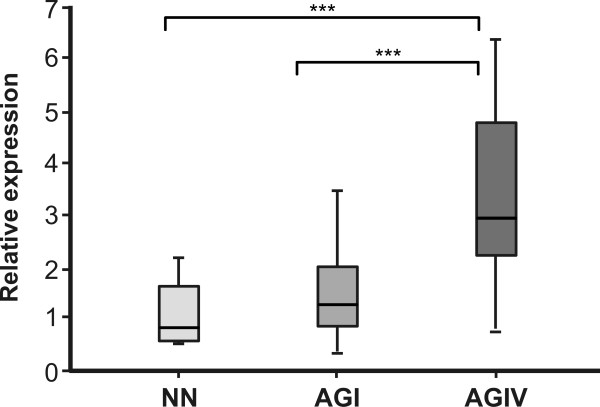
***GRP78 *expression in grade I and grade IV astrocytomas (AGI and AGIV) relative to non-neoplastic (NN) brain tissues. A**) Tissues were reverse transcribed into cDNAs and then analyzed by quantitative Real Time PCR (qRT-PCR) using the SYBR Green method. The geometric mean of the three genes (HPRT, GUSB and TBP) was used for relative expression analysis. The differences of relative *GRP78* expression were statistically significant between groups, ***p = 0.0001. One-way ANOVA and Post hoc Tukey’s test was used to calculate the differences of expression between groups (***p < 0.0001*).* *** p < 0.0001 (NN versus AGI and NN versus AGIV).

**Figure 4:**
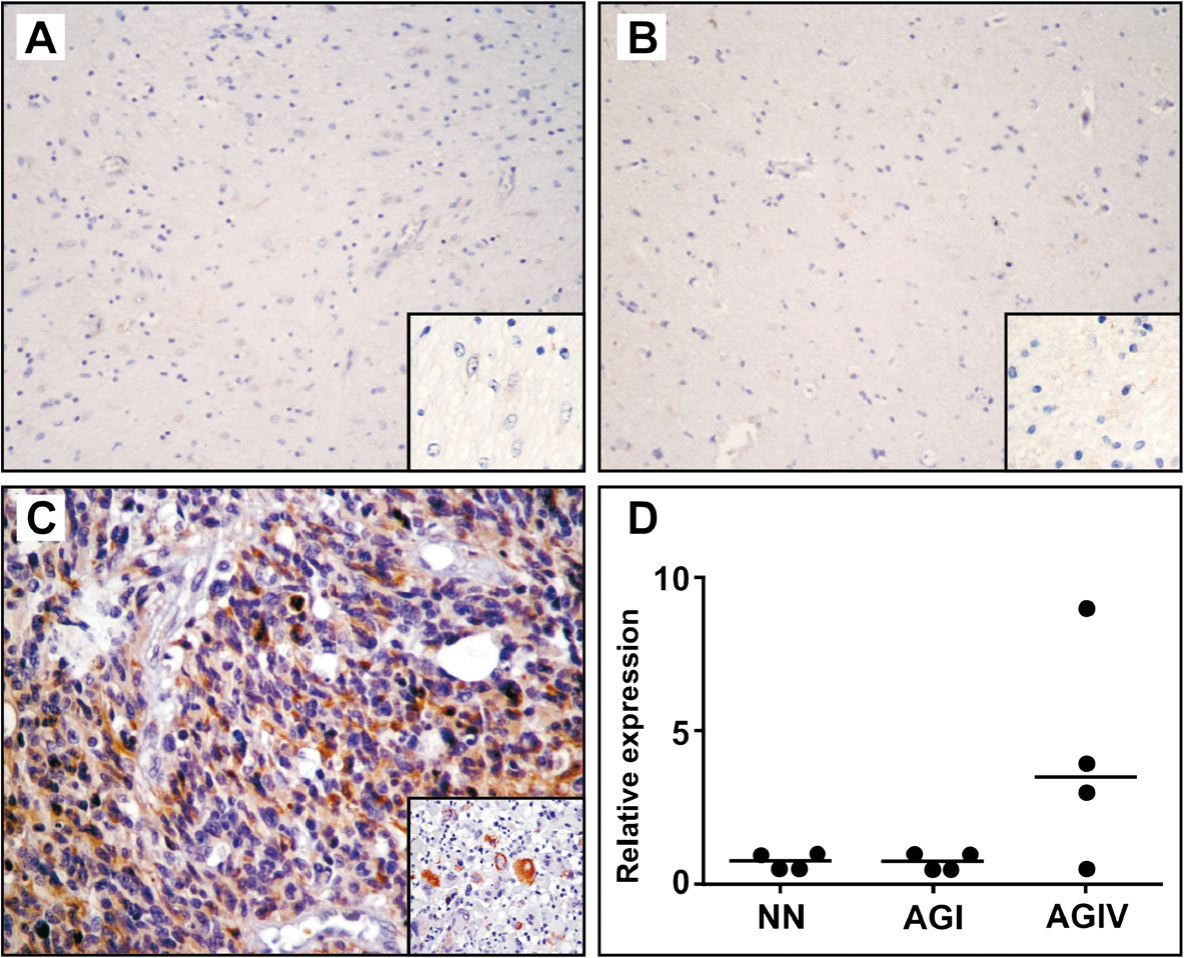
**Immunohistochemical detection of GRP78 in patient tumor samples. A**) Non-neoplastic tissue, **B**) Anaplastic pilocytic astrocytoma grade I, **C**) astrocytoma grade IV and **D**) Scattered graphics of GRP78 relative expression. All immunohistochemical figures are presented at 200x magnification and the figure inserts at 400x magnification. All prepared slides were analyzed independently by two pathologists, and the positive reaction was measured for GRP78 as the percentage of cytoplasm positive cells. Zero (0), when no positivity was detected; 1, when up to 25% of positive cells were present; 2, for 26-50% of positive cells; 3, for 51-75% of positive cells, and 4, for over 76% of positive cells.

In order to further investigate whether the U87MG cell line presented a different cell migration potential compared to T98G, a migration assay was performed by the transwell system using 2.5 × 10^4^ cells/mL (500 μL/well) of each cell line during the first 18 h after plate seeding. As shown in Figure 2B, the migration rate of U87MG cells was higher than that of T98G cells (3100 ± 513 against 260 ± 30, p < 0.0001). This result suggests that the tumorigenic ability of U87MG cells may be due to their late higher proliferation and migration rates. We tested whether cellular stress was involved in this marked cell migration difference and submitted both cell lines to an inducer of endoplasmic reticulum (ER) stress by exposing them to 1 μM thapsigargin (an endogenous calcium releaser), which is known to induce ER stress and to cause an unfolded protein response (UPR) corresponding to the increased expression of GRP78 detected by Western blotting. Accordingly, both cell lines presented a significant decrease of cell migration (Figure 2B) which was inversely correlated to ER stress and to a concomitant increase of GRP78 expression (Figure 2C). To extrapolate this result to human astrocytoma tumors, we probed GRP78 expression by qRT-PCR and immunohistochemistry in grade IV astrocytomas (n = 4) and compared them to grade I tumors (n = 4) and to non-neoplastic tissue (n = 4). Quantitative RT-PCR showed an increased level of *GRP78* mRNA in grade IV astrocytomas (Figure 3). Immunohistochemistry also demonstrated an increased expression of GRP78 at the protein level in grade IV tumors (Figure 4). GRP78 showed a scattered pattern in grade IV astrocytomas by both methods, as opposed to a grouped pattern in grade I tumors and in non-neoplastic tissue.

**Figure 5:**
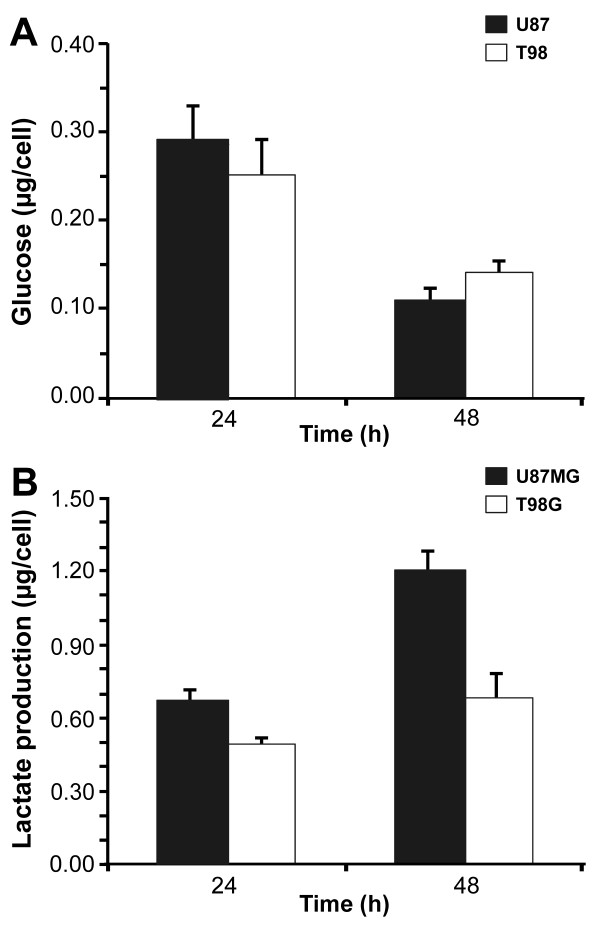
**Quantification of glucose and lactate in T98G and U87MG cell lines under normoxic culture conditions. A**) Quantification of glucose in μg/cell (mean±SD, n = 6, 24 h, p = 0.621 and 48 h, p = 0.0645, Student’s *t*-test). **B**) Quantification of lactate in μg/cell (mean±SD, n = 6, 24 h, p = 0.001 and 48 h p = 0.0005, Student’s *t* test).

Since we found an alteration in the expression of several glycolytic enzymes between these two cell lines, we evaluated glucose consumption and lactate production in six replicate samples for each time and during 24 and 48 h cell growth under normoxic conditions. Glucose quantification indicated no significant difference within 24 h, but U87MG cells tended to consume more glucose at 48 h than T98G cells, expressed as μg glucose/cell (Figure 5A, 24 h, p = 0.621 and 48 h, p = 0.0645, Student’s *t* test). However, there was a higher production of lactate in U87MG cells compared to T98G cells, expressed as μg/cell (Figure 5B, 24 h, p = 0.001 and 48 h p = 0.0005, Student's *t* test). It is noteworthy that, as shown in Figure 2A, cell proliferation differed only after 72 h of culture and the doubling time was very similar for both cell lines under normoxic conditions. Our hypothesis is that U87MG cells can use glucose more efficiently than T98G cells due to a moderate consumption of glucose and a larger production of lactate during cell culture under similar conditions. These results allow us to speculate that U87MG cells may have a greater ability to withstand the initial conditions of hypoxia during tumor formation than T98G cells.

## Discussion

The major difference between these human glioblastoma cell lines, U87MG and T98G, is the tumorigenic potential of U87MG cells in nude mice [6,7]. In fact, our *in vitro* assays showed a higher basal proliferation rate and a higher migration rate of U87MG cells than T98G cells, as reported by others [12-14]. These results partially explain the tumorigenic ability of U87MG cells and the absence of such ability in T98G cells.

In the present study, the proteomic approach used to compare these two cell lines disclosed a differential protein profile that further corroborates the functional differences between them. GRP78 protein expression was four times higher in T98G cells than in U87MG cells. Previous studies on fibroblast cells have shown that thapsigargin and tunicamycin induce UPR and the most significant temporal changes were observed for the 78 kDa glucose-regulated protein (GRP78) [15]. We used thapsigargin to induce UPR and the agent caused an increase of GRP78 expression in both cell lines and a significant decrease in cell migration. Interestingly, migration reduction was much more evident in U87MG than in T98G cells. These results suggest that GRP78 and other proteins altered by the action of thapsigargin as a result of UPR participate in a negative loop for cell migration, and the lower expression of GRP78 in U87MG cells may contribute to the tumorigenic ability of these cells. This observation clearly requires further investigation focusing on GRP78 knockdown by RNA of interference. In agreement with our data, GRP78 silencing increased cell migration in both HepJ5 and Mahlavu cells and overexpressed GRP78 suppressed the migratory ability of skHep1 cells and this effect on GRP78-mediated cell migration was attributed to an increased vimentin expression in hepatocellular carcinoma cells [16,17]. In human brain tumors, we observed that the GRP78 expression at the mRNA and protein level showed a scattered pattern in grade IV astrocytomas, as opposed to a grouped pattern in grade I astrocytomas and in non- neoplastic tissue. This result suggests an important participation of GRP78 in the already well-known heterogeneity of astrocytomas which perhaps could be useful as a prognostic factor.

Metabolic reprogramming is an additional relevant aspect of malignant cells [18]. Activation of the glycolytic pathway even under aerobic conditions (Warburg effect) assures tumor growth, migration and invasion [19]. Cumulative reports have shown that GBM cells are able to increase cell migration and invasion despite the irregular blood supply in a hypoxic microenvironment by activating glycolysis [20,21]. It has also been shown that GBM cells present sufficient glycolytic capacity to support the migration of tumor cells without mitochondrial participation [22-24].

Interestingly, our proteomic data showed that U87MG cells, with a higher migration rate than T98G cells, presented overexpression of several enzymes involved in glycolysis, whereas T98G presented overexpression of glucose-6-phosphate 1-dehydrogenase (G6PD), the enzyme that commands the shift of glycolysis to the pentose phosphate pathway (PPP). It has been recently reported that G6PD is under regulation of cytoplasmic p53 which regulates its biosynthesis by direct inactivation of G6PD [25,26]. Notably, G6PD overexpression was observed in the T98G cell line presenting mutated TP53. Therefore, it is reasonable to speculate that TP53 mutation in this cell line decreases cytoplasmic p53, leading to less inactivation of G6PD, and consequently to attenuated activation of glycolysis because of the shift to PPP. This metabolic reprogramming might ultimately interfere with the ability of the T98G cell line to induce tumors in nude mice.

In summary, the present report provides the proteomic profile of two glioma cell lines with distinct tumorigenic potential, contributing to a better understanding of their functional differences at the protein level and highlights the involvement of GRP78 and proteins involved in UPR in the migration of these two glioma cell lines.

## Conclusions

Taken together, the present results suggest an important role of proteins involved in key functions such as glycolysis and cell migration that may explain the difference in tumorigenic ability between these two glioma cell lines and may be extrapolated to the differential aggressiveness of glioma tumors.

## Material and methods

### Cell culture and tumor samples

GBM cell lines, T98G and U87MG, were maintained at 37°C in 5% CO_2_, in α-MEM medium supplemented with 15% heat-inactivated fetal bovine serum (FBS) and DMEM medium supplemented with 10% FBS, respectively. Cells were cultured up to 80% confluence and were harvested after trypsin treatment. Cells were then washed twice in PBS, pH 7.4, and counted in a Neubauer chamber. An aliquot of the cell pellet containing ~ 1x10^7^ cells was frozen at -80°C for further protein extraction. For thapsigargin treatment, cells were cultured in 35 mm plates with 1 μM thapsigargin in medium for 24 h. The use of human material in this study was approved by the National Bioethics Commission of Brazil and by the Ethics Committee of the Medical School of Sao Paulo, University of Sao Paulo. Written consent was obtained from patients authorizing the use of their tissues in the present investigation.

### Cell proliferation and doubling time assay

Cells were counted in a Neubauer chamber. Briefly, 2 × 10^5^ cells of each line were plated onto 100 mm^2^ plates. Every 24 h the cells were treated with trypsin, centrifuged and resuspended in PBS for counting after dilution in a Turkey’s solution. Two counts were performed for each lineage, in triplicate. For doubling time analysis, cells were plated onto 6-well plates in triplicate at a concentration of 5 × 10^4^ cells/well in DMEM. After 24, 48, 72 and 96 h, cells were collected after trypsinization and counted in a Neubauer chamber. Doubling time (in h) was calculated using the following formula = h × ln(2)/ln(c2/c1), where c is the number of cells at each time of collection and ln is a neperian logarithm. (Roth V, 2006 http://www.doubling-time.com/compute.php).

### Cell migration assay

Transwell assays were done using 24-well plate inserts with pores 8 μm in diameter (Greiner Bio-One), in which 2.5 × 10^4^ cells/mL (500μL/well) were plated on the top of the insert in serum-free medium containing 1 μM thapsigargin. The bottom of the pit was covered with 500 μL of DMEM and α-MEM with 10 or 15% fetal bovine serum (FBS), respectively, for the U87MG and T98G cell lines. Cells were allowed to migrate toward to the bottom chamber containing medium with FBS for 18 h. The cells on the upper membrane surface were removed with a cotton tip, and the migratory cells attached to the lower membrane surface were fixed in 4% paraformaldehyde and stained with 0.2% crystal violet solution. The lower membrane surface was photographed in ten randomly selected fields under 100x microscope magnification and the cells were counted in each field using the Image J software (NIH, Bethesda, MD). The number of migratory cells was obtained by triplicate analysis of three individual wells.

### Protein extraction

T98G and U87MG cell pellets of were thawed at 4°C and resuspended in lysis solution with 7.7 M urea, 2.2 M thiourea, 4% CHAPS and a protease inhibitor cocktail (Sigma-Aldrich, St. Louis, MO), submitted to three cycles of 5 minutes in an ultrasound bath (UltraSonic Clear 750, UNIQUE) for complete pellet resuspension and centrifuged at 20,000 x *g* for 30 min at 4°C. Protein concentration was determined by the method of Bradford [27].

### Two-dimensional gel electrophoresis

The first dimensional is electric focusing (IEF) was done on the IPGphor system (Ettan IPGphor I GE Healthcare, Psicataway, NJ) using Immobiline Dry IPG strips (7 cm, pH 3-10 non-linear; GE Healthcare, Uppsala, Sweden) and 300 μg of protein extract were applied to IPG strips. IEF was started at 500 V and the voltage was gradually increased to 5000 V until completing 40,000 volt-h and the procedure ended at 8000 V for 30 minutes. After IEF, the gel strips were equilibrated in buffer I for reduction (6 M urea; 50 mM Tris–HCl, pH 8.8, 2% SDS, 30% glycerol, and 1% DTT), and buffer II for alkylation (1% DTT was replaced with 2.5% iodoacetamide); each sample was gently shaken for 15 minutes at room temperature (22°C). The second dimensional separation was done on 12.5% SDS-polyacrylamide gels (10 cm × 10 cm × 1 mm) using Hoofer SE 600 vertical chambers. Gels were stained with colloidal Comassie blue-G250 and images were captured by transmission scanning (Image Scanner, Pharmacia-Biotech, Sweden) using Magic Scan software (GE-Healthcare). Gel images were analyzed with the Image Master software, v.7.0 (GE-Healthcare). Replicates of 12 gels (triplicate of gels for each cell line) were obtained, and statistical analysis was carried out using Anova followed by the Student’s *t*-test for the selection of protein spots ( Additional file 2: Table S2).

### Trypsin digestion and mass spectrometry analysis

Selected protein spots were manually excised from the gels. SDS and Coomassie blue were removed by successive changes of 50% acetonitrile in 0.1 M of ammonium bicarbonate, pH 7.8, following dehydration in neat acetonitrile and drying in Speed Vac (Savant, New York, NY, USA). Proteins were then digested with 0.5 μg of modified trypsin (Promega Corp., Madison, WI, USA) in 0.1 M ammonium bicarbonate for 18 h at 37°C. The reaction was stopped by the addition of 1 μL of neat formic acid. Tryptic peptides were extracted by passive elution and desalted in micro-tips filled with reverse-phase resin (POROS R2, Perceptive Biosystems, Foster City, CA, USA), previously equilibrated in 0.2% formic acid. The sample was desalted by two washes of 150 μL of 0.2% formic acid. Peptides were eluted in 30 μL of 60% methanol/5% formic acid solution, concentrated in Speed Vac and resuspended in α-cyano-4-hydroxycinnamic acid matrix solution (5 mg/mL). Two to five micro liters of each sample were loaded in MALDI target and analyzed with a MALDI-TOF-TOF mass spectrometer (Axima Performance – Kratos-Shimadzu, Manchester, UK).

### Protein identification

PMF and CID-MS/MS spectra from 2 DE selected protein spots were submitted to protein identification by searching against the Swiss-Prot database version 57.2 filtered for *Homo sapiens* (total of 20,402 human sequences) using MASCOT version 2.2.04. The database search parameters were as follows: trypsin hydrolysis, one missing cleavage was allowed, fixed modification for carbamidomethyl-Cys and variable modification for methionine oxidation. The mass tolerance for precursor ions was 1.2 Da and mass tolerance for fragment ions was set at 0.8 Da. Protein identification was supported by MS/MS analysis of individual ions using CID-MS/MS. Proteins were identified on the basis of at least two unique peptides or one peptide if its score was higher than 35 (p < 0.05) or the amino acid sequence was consistently covered by a series of *b* and *y* type ion fragments ( Additional file 2: Table S2 and pride XML compatible file as Additional file 3).

### Protein ontology

The differentially expressed proteins were submitted to GO:: Term Finder software [28] and functional classification for biological processes was performed by gene ontology ( Additional file 1).

### Western blot

Western blotting was used to verify the expression of GRP78 in the protein extract from U87MG and T98G cell lines before and after thapsigargin. Samples were submitted to SDS-PAGE (12% polyacrylamide gels) and transferred to Hybond PVDF membranes (Amersham Biosciences) for one h at 35 V. A PVDF membrane was blocked with 5% dry non-fat milk, followed by incubation with goat polyclonal anti-GRP78 antibody (Santa Cruz Biotechnology). After TBS-T washing, PVDF membranes were incubated with donkey anti-goat immunoglobulin-peroxidase conjugate (Sigma Chemical) for 1 h at room temperature (22°C). The immune complexes were visualized by enhanced chemiluminescence using an ECL kit (GE Healthcare), and detected using Image Quant LAS 4000 mini (GE-Healthcare, USA).

### Quantification of glucose and lactate under normoxic cell culture conditions

U87MG and T98G cell lines were seeded onto six well plates and incubated for 24 and 48 h with 3.5 × 10^4^ cells (10 cm^2^/well) containing 2 mL of culture medium (4 g/L glucose, 2.6 g/L HEPES, 3.6 g/L NaHCO_3_, 1% penicillin/streptomycin, 1% L-glutamine, 10% FBS). In order to measure glucose consumption and lactate formation by U87MG and T98G cells, 3.5 × 10^4^ cells were incubated in six-well plates. After a stabilization period, the plates were washed using phosphate buffered saline (PBS) and the medium was replaced with 2 mL of phenol-red-free minimum medium containing glucose, fetal calf serum (FCS) and L-glutamine. The assays were performed 24 and 48 h after incubation, along with cell counting. For glucose and lactate quantification, 200 μL supernatant samples were collected in micro tubes and frozen until the time for assay. Glucose quantification was performed using an enzymatic colorimetric assay based on the D-glucose oxidase method (Latest Diagnostic, São Paulo, Brazil). The color intensity was measured at 520 nm. The D-Lactate Assay Kit (Latest Diagnostic, São Paulo, Brazil) was used to measure D-lactate which is specifically oxidized by D-lactate oxidase and generates proportional color (λmax = 550 nm). The protocols were developed according to manufacturer instructions. Data were reported as glucose and lactate in μg/cell and represent the mean ± SEM of 6 determinations, and were analyzed by the Student’s *t* test.

### Total RNA extraction and cDNA synthesis

Total RNA was extracted from tissues using RNeasy Mini Kit (Qiagen Inc, Hilden, Germany). RNA quantification and purification was evaluated by measuring absorbance and A260/A280 ratios in the range of 1.8-2.0 were considered satisfactory for purity standards. Denaturing agarose gel electrophoresis was used to assess the quality of the samples. Synthesis of cDNA was performed by reverse transcription from 1 μg total RNA, previously treated with 1 unit of DNase I (FPLC-pure, GE Healthcare, Piscataway, NJ) using random and oligo(dT) primers, an RNase inhibitor and Superscript III (Invitrogen Inc, Carlsbad, CA), following the manufacturer’s recommendations. The resulting cDNA was then treated with 1 unit of RNase H (GE Healthcare), diluted with TE buffer, and stored at -20°C until later use.

### Quantitative real time (qRT-PCR)

The expression level of *GRP78* was determined by qRT-PCR using the SYBR Green approach, in duplicate. Quantitative data were normalized relative to the internal housekeeping genes hypoxanthine phosphoribosyltransferase 1 (*HPRT*), beta-glucuronidase (*GUSB*) and TATA-box binding protein (*TBP*). The geometric mean of the three genes was used for relative expression analysis. Primer sequences were as follows (5′-3′): GRP78 F: GGTGACCTGGTACTGCTTGATGT, GRP78 R: TCCTTGGAATCAGTTTGGTCAT, HPRT F: TGAGGATTTGGAAAGGGTGT, HPRT R: GAGCACACAGAGGGCTACAA, GUS*B* F: AAAATACGTGGTTGGAGAGCTCATT, GUSB R: CCGAGTGAAGATCCCCTTTTTA, TBP F: AGGATAAGAGAGCCACGAACCA and TBP R: CTTGCTGCCAGTCTGGACTGT synthesized by IDT (Integrated DNA Technologies, Coralville, IA). Sybr Green I amplification mixtures (12 μl) contained 3 μl of cDNA, 6 μl of 2x Power Sybr Green I Master Mix (Applied Bios stems, Foster City, CA), and forward and reverse primers. Reactions were run on an ABI 7500 Real-Time PCR System (Applied Bios stems). The cycle conditions comprised incubation at 50°C for 2 min to activate UNG, initial denaturation at 95°C for 10 min, and 40 cycles at 95°C for 15 sec, and 60°C for 1 min. The minimum concentration of primers was determined by the lowest threshold cycle (Ct) and maximum amplification efficiency while minimizing nonspecific amplification (at a final concentration of 200 nM for *GRP78*, *HPRT* and *TBP*, and of 400 nM for *GUSB*). Analysis of DNA melting curves demonstrated a single peak for the whole set of primers. Standard curves were analyzed for all genes to check the efficiency of amplification of each gene. Additionally, agarose gel electrophoresis was employed to check the size of the amplified PCR product. *GRP78* relative expression levels were analyzed in 10 non-neoplastic brain tissues (NN), 10 grade I astrocytomas (AGI) and 20 grade IV astrocytomas (AGIV). The 2^-ΔΔCT^ equation was applied to calculate the relative expression of tumor samples versus the mean of non-neoplastic tissues where ΔCt = mean Ct *GRP78* – Ct geometric mean of housekeeping genes and ΔΔCt = ΔCt tumor – mean ΔCt non-neoplastic tissues [29].

### Immunohistochemistry

For immunohistochemical detection of GRP78, tissue sections were routinely processed and subjected to antigen retrieval. Briefly, slides were immersed in 10 mM citrate buffer, pH 6.0 and incubated at 122°C for 3 min using an electric pressure cooker (BioCare Medical Walnut Creek, CA). Specimens were then blocked and further incubated with a mouse monoclonal antibody raised against human GRP78 (goat polyclonal, N-20, Santa Cruz – sc-1050) at a final dilution of 1:200 at 16-20°C for 16 h. The reaction was developed using a Novolink commercial kit (Novocastra, New Castle, UK) at room temperature using diaminobenzidine, and Harris hematoxylin for nuclear staining. All prepared slides were independently analyzed by two pathologists, and the positive reaction was quantitated for GRP78 as the percentage of positive cytoplasm cells: zero (0), when no positivity was detected; 1, when up to 25% of positive cells were present; 2, for 26-50% of positive cells; 3, for 51-75% of positive cells, and 4, for over 76% of positive cells.

### Statistical analysis

The statistical analysis of *GRP78* expression by qRT-PCR in AGI, AGIV samples and NN tissues was performed by one-way ANOVA. Post hoc Tukey’s tests were applied to compare the differences between the non-neoplastic group and each tumor group Differences were considered to be statistically significant at *p* < 0.05.

## Competing interests

The authors declare that they have no competing interest.

## Authors' contributions

AR carried out the proteomic studies, participated in the experimental design and drafted the manuscript. MG carried out the 2 D Gel image and statistical analysis. HLJ provided mass spectrometry analysis and revised the manuscript. CI provided mass spectrometry analysis and revised the manuscript, RCV carried out the metabolic assay experiment and revised the manuscript, SOS and SKNM carried out molecular biology analysis and immunohistochemistry, and drafted the manuscript, JCR conceived the study and participated in its design and coordination and also drafted the manuscript. All authors read and approved the final manuscript.

## Supplementary Material

Additional file 1 Protein Ontology (GO-TermFinder). Click here for file

Additional file 2 **Table S1. **Supplemental data of protein identification by MALDI-TOF/TOF-MS of tryptic peptides obtained from 2-DE spots of T98G and U87MG glioma cell lines. Click here for file

Additional file 3 mascotDatFile_Ramao-U87MG_and_T98G_to_PRIDE_20120619_213931.xml. Click here for file
